# Phytochemical Investigation of *Vinca minor* Cultivated in Iran

**Published:** 2011

**Authors:** Behnaz Farahanikia, Tahmineh Akbarzadeh, Akbar Jahangirzadeh, Narguess Yassa, Mohammad Reza Shams Ardekani, Tahmineh Mirnezami, Abbas Hadjiakhoondi, Mahnaz Khanavi

**Affiliations:** a *Department of Pharmacognosy and Medicinal Plants Research Center, Faculty of Pharmacy, Tehran University of Medical Sciences, Tehran, Iran.*; b *Department of Medicinal Chemistry, Faculty of Pharmacy, Tehran University of Medical Sciences, Tehran, Iran.*

**Keywords:** *Vinca minor*, Apocynaceae, Indole alkaloids, Vincamine

## Abstract

Lesser Periwinkle (*Vinca minor *L.), a member of Apocynaceae, is not only an ornamental plant with lilac-blue flowers, but also a medical plant producing an important alkaloid, vincamine, found in the leaves which shows a pronounced cerebrovasodilatory and neuroprotective activity. This plant is native to northern Spain, western France, central and southern Europe, and Caucasus. It has been recently cultivated for pharmaceutical purposes by Zardband Botanical Garden in Iran. Since the quality of herb material and alkaloid concentration is greatly influenced by environmental conditions, in this study, we report the isolation and identification of major alkaloids along with the quantification of vincamine as the pharmacologically most important component.

Alkaloids from the aerial parts of *V. minor *were isolated and purified using different chromatographic methods. The structures of these alkaloids were determined on the basis of their physical and spectroscopic data. The concentration of vincamine was determined by high performance liquid chromatography using Tracer Excel 120 ODS A C18 column.

Five indole alkaloids including vincaminorine, vincaminoreine, minovine, minovincine, and vincamine (Figure 1) were isolated from the aerial parts of *V. minor*. Vincamine was found to be the dominant alkaloid in this plant with the content of 0.057% of the dried plant mass.

This plant may be used as a natural source for pharmaceutical purposes in Iran, due to the presence of biologically active alkaloids especially vincamine as the major alkaloid in Lesser Periwinkle cultivated.

## Introduction

The genus *Vinca *(Apocynaceae) comprises about seven species in the world. In Iran, it is represented by *V. herbacea *Waldst and Kit as a native plant with two other species being introduced or cultivated including *V. minor *L. and *V. major *L. (1, 2). *Vinca minor *or Lesser Periwinkle is a perennial subshrub, indigenous to northern Spain, through western France, eastward via central and southern Europe as far as the Caucasus; it has been naturalized in many regions. In folk medicine, it is used internally for circulatory disorders, cerebral circulatory impairment and brain’s metabolism support (3).

So far, more than 50 alkaloids of indole type have been isolated from the aerial parts and the root of this plant, a few of which have quaternary structures such as 4-methylraucubaininium chloride, 4-methylstrictaminium chloride and 4-methylakuammicinium chloride (4).

**Figure 1:**
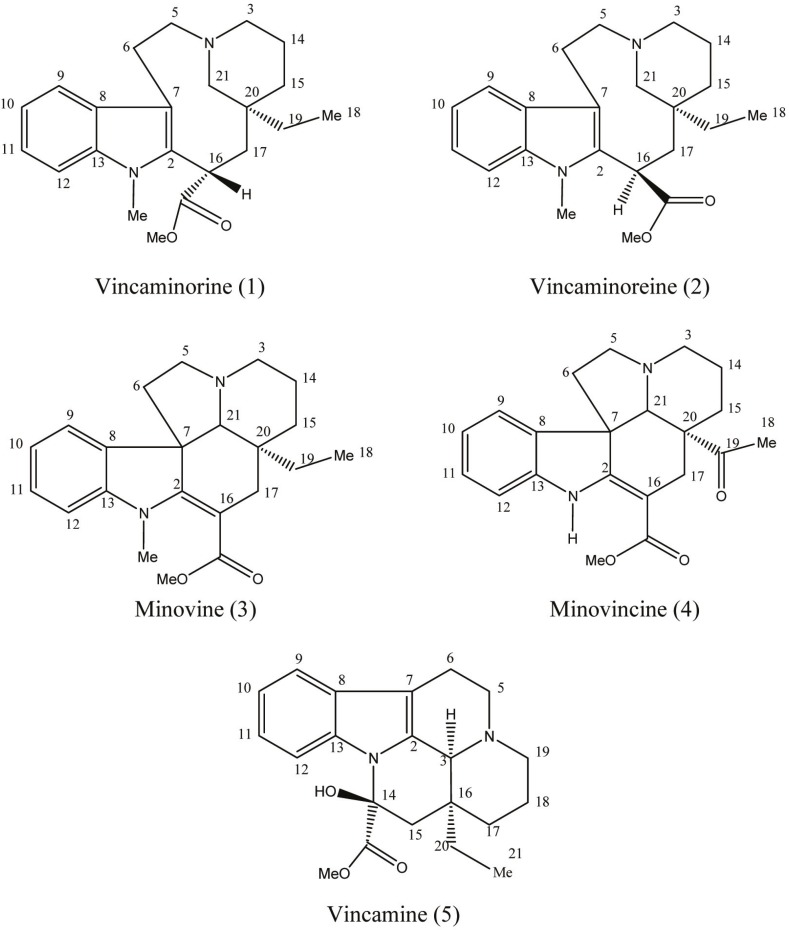
Structures of alkaloids isolated from the aerial parts of *Vinca minor *(compounds 1-5).

*Vincarubine is the only alkaloid with bisindole structure reported from this plant that has shown a considerable cytotoxic effect against the P388 leukemia cells (* 5 *). *V.minor *contains monomeric eburnamine-type indole alkaloids including vincamine which has modulatory effects on brain circulation and neuronal homeostasis as well as antihypoxic and neuroprotective potencies (*6*). Vincamine is used for the prevention and treatment of cerebrovascular insufficiencies and disorders. A large body of clinical evidence indicates a favorable effect of vincamine in a number of brain disorders of elderly patients, such as memory disturbances, vertigo, transient ischemic deficits, and headache. It increases cerebral blood flow, oxygen consumption and glucose utilization (*7*, *8*). In general, *Vinca *minor *is known to be a valuable medicinal plant in folk medicine and it also serves as a natural source for the industrial production of medicaments for stimulating the brain blood flow (9). *Vinca minor *has been recently cultivated by Zardband Botanical Garden in Gonbad Qabus, Golestan Province, in large scale for pharmaceutical purposes. In view of the fact that different parameters including climate and soil requirements, agricultural measures such as propagation, planting, fertilization and harvest date vastly influence the quality of plant material and alkaloid concentration, the analysis of the alkaloid content of the plant grown in Iran proves invaluable. It has been shown that nitrogenous fertilizers significantly increase the content of alkaloids. Besides, the content of alkaloids reaches its maximum at the flowering stage (May to September). Proper air temperature and humidity as well as sufficient soil moisture during growth and development of the plant are important factors for growing and dry matter production of *Vinca minor *(10, 11). All of the aforementioned factors should be taken into consideration in order to optimize the growth and alkaloid yield of the herb. We have previously reported the cytotoxicity of the alkaloid fractions of this plant (12). This is the first phytochemical report of *Vinca minor *cultivated in Iran. We report here the isolation and characterization of alkaloids vincaminorine, vincaminoreine, minovine, minovincine, and vincamine along with a validated HPLC quantification of vincamine (Figure 1).

## Experimental

*General*

Melting points (uncorrected) were determined on a Reichert-Jung apparatus. Optical rotations were measured in 2 cm cells on a Perkin-Elmer 142 automatic spectropolarimeter. FT-IR spectra were recorded on a Nicolet 550-A spectrometer (KBr pellets). EI-MS spectra were measured by Agilent Technology (HPTM) instrument with 5973 Network Mass Selective Detector (MS model). ^1^H- and ^13^C-NMR spectra of CDCl3 solution, running with a Bruker Avance DRX 500 spectrometer operating at 500 and 125 MHz, respectively, are relative to TMS as an internal standard. Chemical shifts (*δ*) are expressed in ppm and coupling constants (*J*) in Hz. HPLC analysis was performed using a Waters Alliance system. The column used was Tracer Excel 120 ODS A C18 column (150 × 5 mm, 5 μm). Merck silica gel 60 (70-230 mesh), Merck silica gel 60 (230-400 mesh), Merck TLC silica gel 60 F_254_ aluminium sheets (20 × 20 cm) and Merck silica gel 60 GF_254_ were used for column chromatography, medium pressure liquid chromatography (MPLC), analytical and preparative TLC, respectively. With the exception of vincamine standard which was obtained from Sigma-Aldrich, all other chemicals were purchased from Merck Company.

Alkaloid spots on chromatograms were detected under UV-light or by spraying with Dragendroff›s reagent.

*Plant material*

The aerial parts of *V. minor *were collected from Zardband Botanical Garden in Gonbad Qabus, Golestan Province, in June 2006. The plant was identified and authenticated by Dr. Gholamreza Amin and the voucher specimen (TEH-6654) was deposited at the Herbarium of the Faculty of Pharmacy in Tehran University of Medical Sciences (Tehran, Iran).

*Extraction and isolation*

The air-dried and powdered aerial parts of the plant (800 g) were extracted with 80% MeOH at room temperature for 72 h. The procedure was repeated until the negative test against Dragendroff›s reagent. The MeOH extracts were concentrated to give 287 g of crude extract (yield: 35.87%). The crude extract was dissolved in CHCl_3_ (500 mL) and extracted with 2N HCl (200 mL × 10). The acidic fraction was washed with CHCl_3 _(200 mL × 3). The pH of the aqueous solution was adjusted to 2. The combined acid fraction was basified with 25% NH_3_ on the ice chest (pH 10-12) and extracted with CHCl_3_ (300 mL × 10). CHCl_3 _was removed under reduced pressure to give 10.9 g of a crude mixture of alkaloids. A mass of 3 g crude alkaloid extract was initially subjected to column chromatography on silica gel (70-230 mesh), eluted with CHCl_3_-MeOH in a step gradient (99:1, 98:2, 97:3, 96:4, 95:5, 90:10, 80:20, 70:30, 60:40, 50:50, 25:75, and 10:90 v/v; 500 mL each) to give twelve fractions. Fractions with similar TLC behavior were combined to yield the following three major fractions: 1 (0.72 g), 2 (0.85 g) and 3 (0.97 g).

Fraction 1 was purified using a medium pressure liquid chromatography (MPLC) over silica gel (230-400 mesh) with the solvent system of Hexane-EtOAc (8:2) to give 4.2 mg of vincaminorine (1), 4.5 mg of vincaminoreine (2), 12.5 mg of minovine (3), and 6 mg of minovincine (4).

Fraction 2 was subjected to repeated prep. TLC (20 × 20 cm, 0.8 mm thickness) over silica gel eluted with EtOAc-MeOH (9:1) to give 80 mg of vincamine (compound 5) (R:0.40).

*Vincaminorine (compound 1)*

White crystals, m.p. 130–131°C. [*α*]_D_ + 48° (EtOH). IR (KBr) *ν*_max_ cm^-1^: 3025, 2960, 1717, 1690, 1463, 1374, 1258, 1090, 1018, 769. EI-MS 70 eV, *m/z *(rel. int.): 354 (43, M^+^), 339 (1), 323 (2), 295 (3), 268 (3), 229 (100), 210 (6), 184 (3), 170 (5), 149 (6), 124 (17). ^1^H-NMR (500 MHz, CDCl_3_) δ: 7.51 (d, *J *= 7.1, 1H, H-9), 7.26 (1H, covered by solvent peak, H-12), 7.18 (t, *J *= 7, 1H, H-10), 7.09 (t, *J *= 7, 1H, H-11), 6.22 (m, 1H, H-16), 3.65 (s, 3H, OMe), 3.61 (s, 3H, NMe), 2.91–2.93 (m, 3H, CH_2_-14, H-3a), 2.62–2.65 (m, 1H, H-5a), 2.42–2.44 (m, 1H, H-3b), 2.39–2.42 (m, 2H, CH_2_-17), 2.33–2.39 (m, 1H, H-5b), 2.22 (d, *J *= 15, 1H, H-21a), 1.83 (d, *J *= 15, 1H, H-21b), 1.60 –1.62 (m, 1H, H-6a), 1.25–1.29 (m, 2H, CH_2_-19), 1.07–1.10 (m, 1H, H-6b), 1.03–1.06 (m, 1H, H-15a), 0.97–1.03 (m, 1H, H-15b), 0.66 (t, *J *= 7, 3H, Me-18). ^13^C-NMR (125 MHz, CDCl_3_) δ: 176.36 (CO), 138.75 (C-2), 138.25 (C-13), 128.75 (C-8), 122.40 (C-10), 120.13 (C-11), 119.61 (C-9), 114.00 (C-7), 110.10 (C-12), 62.26 (C-21), 55.52 (C-5), 55.00 (C-3), 53.58 (OMe), 40.36 (C-17), 40.29 (C-16), 37.13 (C-6), 36.47 (C-15), 32.28 (NMe), 28.10 (C-14), 26.32 (C-20), 15.59 (C-19), 8.84 (C-18) (13).

*Vincaminoreine (compound 2)*

White crystals, m.p. 126°C. [*α*]_D_ + 26.5° (CHCl_3_). IR (KBr) *ν*_max_ cm^-1^: 3016, 2772, 1760, 1568, 1519, 1491, 1342, 1225, 1073, 760. EI-MS 70 eV, *m/z *(rel. int.): 354 (83, M^+^), 339 (3), 325 (3), 295 (8), 268 (9), 229 (100), 210 (60), 184 (13), 170 (19), 149 (9), 124 (43). ^1^H-NMR (500 MHz, CDCl_3_) δ: 7.50 (d, *J *= 7.1, 1H, H-9), 7.24 (1H, covered by solvent peak, H-12), 7.17 (t, *J *= 7, 1H, H-10), 7.07 (t, *J *= 7, 1H, H-11), 3.70 (s, 3H, OMe), 3.52 (s, 3H, NMe), 3.16 (d, *J *= 15, 1H, H-21a), 2.93–2.95 (m, 2H, CH_2_-17), 2.49–2.51 (m, 1H, H-3a), 2.29–2.33 (m, 1H, H-5a), 2.27–2.29 (m, 1H, H-5b), 2.24–2.27 (m, 1H, H-3b), 1.93–1.96 (m, 1H, H-16), 1.61 (d, *J *= 15, 1H, H-21b), 1.47 – 1.54 (m, 1H, H-14a), 1.36–1.41 (m, 1H, H-6a), 1.30–1.35 (m.1H, H-15a), 1.25–1.29 (m, 1H, H-14b), 1.20–1.25 (m, 2H, CH_2_-19), 1.18 – 1.20 (m, 1H, H-15b), 1.16 – 1.18 (m, 1H, H-6b), 0.93 (t, *J *= 7.2, 3H, Me-18). ^13^C-NMR (125 MHz, CDCl_3_) δ: 175.17 (CO), 139.27 (C-2), 136.74 (C-13), 127.05 (C-8), 120.59 (C-10), 118.49 (C-11), 117.96 (C-9), 109.96 (C-7), 108.59 (C-12), 57.88 (C-21), 55.27 (C-5), 53.29 (C-3), 52.48 (OMe), 34.21 (C-6), 33.79 (C-16), 31.01 (C-15), 30.23 (NMe), 24.88 (C-20), 22.45 (C-17), 22.32 (C-14), 14.19 (C-19), 7.39 (C-18) (13).

*Minovine (compound 3)*

White amorphous substance, m.p. 79–81°C. IR (KBr) *ν*_max_ cm^-1^: 3016, 2933, 2854, 2776, 1737, 1676, 1585, 1486, 1433, 1374, 1301, 1215, 759. EI-MS 70 eV, *m/z *(rel. int.): 352 (40), 337 (1), 321 (3), 293 (2), 267 (4), 252 (4), 228 (5), 207 (3), 182 (4), 168 (9), 124 (100). ^1^H-NMR (500 MHz, CDCl_3_) δ: 7.20–7.23 (m, 1H, H-11), 7.18 – 7.20 (m, 1H, H-9), 6.91 (t, *J *= 5, 1H, H-10), 6.82 (d, *J *= 7, 1H, H-12), 3.75 (s, 3H, OMe), 3.24 (s, 3H, NMe), 3.11–3.13 (m, 1H, H-3a), 2.92 (d, *J *= 12.5, 1H, H-17a), 2.87–2.91 (m, 1H, H-5a), 2.60 (s, 1H, H-21), 2.48 (d, *J *= 12.5, 1H, H-17b), 2.45–2.47 (m, 1H, H-5b), 2.41–2.45 (m, 1H, H-3b), 1.97–2.00 (m, 1H, H-6a), 1.82–1.86 (m, 1H, H-15a), 1.79–1.82 (m, 1H, H-14a), 1.63–1.65 (m, 1H, H-6b), 1.51–1.53 (m, 1H, H-14b), 1.20–1.22 (m, 1H, H-15b), 0.87–0.89 (m, 1H, H-19a), 0.65–0.69 (m, 3H, Me-18), 0.62–0.65 (m, 1H, H-19b). ^13^C-NMR (125 MHz, CDCl_3_) δ: 168.00 (CO), 167 (C-2), 146.88 (C-13), 138.59 (C-8), 127.55 (C-11), 121.00 (C-9), 120.82 (C-10), 108.48 (C-12), 92.80 (C-16), 75.37 (C-21), 56.76 (C-7), 52.04 (C-5), 50.94 (OMe), 50.40 (C-3), 46.98 (C-6), 37.26 (C-20), 36.17 (NMe), 32.47 (C-15), 30.25 (C-17), 29.33 (C-19), 22.67 (C-14), 7.00 (C-18) (14).

*Minovincine (compound 4)*

White amorphous substance, m.p. 141–142°C. IR (KBr) *ν*_max_ cm^-1^: 3398, 3025, 2932, 2854, 1739, 1708, 1603, 1468, 1440, 1250, 749. EI-MS 70 eV, *m/z *(rel. int.): 353(12), 352 (45, M^+^), 337 (3), 321 (4), 309 (33), 293 (5), 265 (8), 249 (6), 214 (32), 206 (9), 139 (34), 138 (100). ^1^H-NMR (500 MHz, CDCl_3_) δ: 8.80 (br s, 1H, NH), 7.34 (d, *J *= 7.3, 1H, H-9), 7.16 (dt, *J *= 7.6, 1.2, 1H, H-11), 6.96 (t, *J *= 7.7, 1H, H-10), 6.81 (d, *J *= 7.7, 1H, H-12), 3.81 (s, 3H, OMe), 3.30 (d, *J *= 1.2, 1H, H-21), 3.16–3.18 (m, 1H, H-5a), 3.09 (d, *J *= 15, 1H, H-17a), 2.99–3.01 (m, 1H, H-3a), 2.84 (d, *J *= 15, 1H, H-17b), 2.74 – 2.79 (m, 1H, H-5b), 2.49 – 2.54 (m, 1H, H-3b), 1.98 – 2.03 (m, 3H, CH_2_-14 and H-15b), 1.92 (s, 3H, Me-18), 1.79–1.82 (m, 1H, H-6a), 1.65–1.69 (m, 1H, H-15b), 1.46–1.50 (m, 1H, H-6b). ^13^C-NMR (125 MHz, CDCl_3_) δ: 212.50 (C-19), 168.75 (CO), 168.65 (C-2), 142.80 (C-13), 138.60 (C-8), 127.64 (C-9), 121.60 (C-11), 121.11 (C-10), 109.86 (C-12), 91.89 (C-16), 68.13 (C-21), 56.65 (C-7), 54.35 (C-5), 51.91 (OMe), 51.52 (C-3), 50.22 (C-6), 45.70 (C-20), 31.53 (C-17), 26.29 (C-15), 25.50 (C-14), 22.79 (C-18) (15, 16).

*Vincamine (compound 5)*

White crystals, m.p. 230–233°C. IR (KBr) *ν*_max_ cm^-1^: 3430, 3053, 2950, 2927, 1748, 1615, 1457, 1209, 742. EI-MS 70 eV, *m/z *(rel. int.): 354 (100, M^+^), 339 (16), 325 (14), 307 (31), 295 (45), 284 (13), 267 (51), 252 (87), 237 (27), 224 (40), 209 (17), 180 (22), 167 (27), 149 (13), 133 (13), 115 (11). ^1^H-NMR (500 MHz, CDCl_3_) δ: 7.47–7.49 (m, 1H, H-9), 7.09 – 7.13 (m, 3H, H-10. H-11 and H-12), 4.57 (s, 1H, 14-OH), 3.92 (s, 1H, H-3), 3.82 (s, 3H, OMe), 3.32–3.35 (m, 1H, H-5a), 3.28–3.31 (m, 1H, H-5b), 2.95–3.02 (m, 1H, H-6a), 2.58 – 2.63 (m, 1H, H-6b), 2.54–2.57 (m, 1H, H-19a), 2.49–2.51 (m, 1H, H-19b), 2.25–2.27 (m, 1H, H-20a), 2.22 (d, *J *= 15.1, 1H, H-15a), 2.12 (d, *J *= 15.1, 1H, H-15b), 1.70–1.75 (m, 1H, H-17a), 1.66–1.69 (m, 1H, H-18a), 1.47–1.50 (m, 1H, H-20b), 1.42–1.46 (m, 1H, H-17b), 1.35 –1.38 (m, 1H, H-18b), 0.91 (t, *J *= 7.2, 3H, Me-21). ^13^C-NMR (125 MHz, CDCl_3_) δ: 174.89 (CO), 134.56 (C-13), 132.89 (C-8), 129.39 (C-2), 122.13 (C-10), 120.71 (C-12), 118.92 (C-9), 110.75 (C-11), 106.34 (C-7), 82.32 (C-14), 59.59 (C-3), 54.73 (OMe), 51.40 (C-5), 45.01 (C-19), 44.83 (C-15), 35.56 (C-16), 29.32 (C-20), 25.51 (C-18), 21.16 (C-17), 17.25 (C-6), 8.01 (C-21) (17, 18).

*HPLC analysis*

Chromatographic analysis was performed using a Tracer Excel 120 ODS A C18 column (150 × 5 mm, 5 μm). The mobile phase was an isocratic combination of MeOH : water (75 : 25) (0.02 mol/L NH_4_Cl, pH = 8.2 adjusted with Et3N) and the flow rate was adjusted to 0.5 mL/min. The detector wavelength was 254 nm and the injection volume was 50 μL. The quantification was carried out by an external standard method. Standard vincamine was purchased from Sigma-Aldrich. Calibration curve was obtained using solutions containing vincamine in concentrations of 50, 100 and 200 μg/mL in MeOH, 3 injections per concentration. A linear relationship between peak area and alkaloid concentration was observed with the regression coefficient *r *better than 0.9997 for vincamine standard.

*Sample preparation*

A mass of 5 g of dried ground plant material was extracted in a Soxhlet apparatus with MeOH (50 mL) for 4 h. The solvent was evaporated and the residue was dissolved in CHCl_3_ (30 mL) and extracted with aqueous 2N HCl (4 ×15 mL). The pH of this solution was adjusted to 10 with 25% NH_3_ and the alkaloids were taken up in CHCl_3 _(4 ×15 mL). The combined CHCl_3_ extracts were concentrated, the residue was dissolved in MeOH and the solution was brought to a final volume of 25 mL in a volumetric flask. The solution was filtered and 50 μL of it was injected onto the HPLC column for 3 times.

*Limit of detection and limit of quantization*

Limits of detection (LODs) and limits of quantization (LOQs) were calculated using the expressions 3.3 σ/*s *and 10 σ/*s, *respectively, in which σ is intercept standard deviation and *s *is the slope of calibration curve (19). 

*Selectivity*

For chromatographic method, developing a separation involves demonstrating specificity which is the ability of the method to accurately measure the analyte response in the presence of all interferences. Therefore, the solutions obtained from sample preparation were analyzed and the peaks of analyte were evaluated for peak purity and resolution from the nearest eluting peak.

*Precision*

The precision of each method indicates the degree of dispersion within a series of determinations of the same sample. Three samples in three levels (80, 100. 120%) were analyzed on the same day (intra-day) for three consecutive days (inter-day) and the relative standard deviations (RSD%) were calculated. Three samples for each level were prepared, each of which was injected to HPLC for three times.

*Recovery*

This parameter shows the proximity between the experimental values and the real ones. It ensures that no loss or uptake occurred during the process. The determination of this parameter was performed for the method by studying the recovery after a standard addition procedure with two additional levels. The concentrations of standards added to the sample were 50 and 100 μg/mL. In each additional level, three determinations were carried out and the recovery percentage was calculated in every case. Each sample was injected to HPLC three times.

## Results and Discussion

The alkaloid content of the aerial parts of *V. minor *cultivated in Iran was 1.36%. From the crude alkaloid mixtures, five indole alkaloids were identified and isolated in a pure state. They are vincaminorine (compound 1), vincaminoreine (compound 2), minovine **(**compound 3), minovincine (compound 4), and vincamine (compound 5) (the major alkaloid). Their structures were confirmed by comparison of their spectroscopic data with literature.

Vincaminorine was isolated as white crystals, m.p. 130 – 131°C [*α*]_D_ + 48° (EtOH). The IR spectrum showed absorptions at 1717 (ester C=O), 1690 (C=C), and 769 (1, 2-disubstituted benzene ring). The EI-MS showed a molecular ion at *m/z *354 and other fragment ion peaks at 339 [M^+^-CH_3_], 323 [M^+^-OCH_3_], 295 [M^+^-COOCH_3_], 268, 229, 210, 184, 170, 149 and 124. The ^1^H-NMR showed four aromatic H-atoms. The ester methyl protons resonated at δ3.65 as a 3H singlet. A three-proton singlet at δ3.61 was attributed to N-CH_3_. A peak at δ6.22 representing one proton is due to the H-16 being strongly deshielded by N-4, which makes an outstanding difference between vincaminorine and vincaminoreine ^1^H-NMR spectra. Another difference between vincaminorine and its C-16 epimer is due to their specific rotations [*α*]. The H-21a appeared at δ1.83 as a doublet (*J *= 15 Hz) while another doublet (*J *= 15 Hz) at δ2.22 was assigned to the H-21b. The ^13^C-NMR gave a total of 22 carbon resonances. Ester C=O appeared at δ176.36.

Vincaminoreine was isolated as white crystals, m.p. 126°C [*α*]_D_ + 26.5° (CHCl_3_). The IR spectrum showed absorptions at 1760 (ester C=O), 1568 (C=C), and 760 (1, 2-disubstituted benzene ring). The EI-MS showed a molecular ion at *m/z *354 with further ion fragments of 339 [M^+^-CH_3_], 325 [M^+^-C_2_H_5_], 295 [M^+^-COOCH_3_], 268, 229, 210, 184, 170, 149 and 124. The ^1^H-NMR showed four aromatic H-atoms. The ester methyl protons resonated at δ3.70 as a 3H singlet. A three-proton singlet at δ3.52 was attributed to N-CH_3_. H-16 resonated at δ 1.93-1.96. The H-21a appeared at δ1.61 as a doublet (*J *= 15 Hz) while another doublet (*J *= 15 Hz) at δ3.16 was assigned to the H-21b. The ^13^C-NMR gave a total of 22 carbon resonances. Ester C=O appeared at δ175.17.

Minovine was isolated as white amorphous substance, m.p. 79 – 81°C. The IR spectrum showed absorption bands at 1737 (ester C=O), 1585 (C=C), and 759 (1, 2-disubstituted benzene ring). The EI-MS showed a molecular ion at *m/z *352 with further ion fragments of 337 [M^+^-CH_3_], 321 [M^+^-OCH_3_], 293 [M^+^-COOCH_3_], 267, 252, 228, 207, 182, 168 and 124. The ^1^H-NMR showed the presence of four aromatic protons. The ester methyl protons resonated at δ3.75 as a 3H singlet. One three-proton singlet at δ3.24 was assigned to N-CH_3_. A one-proton singlet at δ2.60 was attributed to H-21. The H-17a appeared at δ2.92 as a doublet (*J *= 12.5 Hz) while another doublet (*J *= 12.5 Hz) at δ2.48 was assigned to the H-17b. The ^13^C-NMR gave a total of 22 carbon resonances. Ester C=O appeared at δ168.

Minovincine was isolated as white amorphous substance, m.p. 141–142°C. The IR spectrum showed intense absorption at 3398 (N-H) along with other absorption bands at 1739 (ester C=O), 1708 (ketone C=O), 1603 (C=C), and 749 (1, 2-disubstituted benzene ring). The EI-MS showed a molecular ion at *m/z *352 with other ion fragments of 337 [M^+^-CH3], 321 [M^+^-OCH_3_], 309 [M^+^-COCH_3_], 293 [M^+^-COOCH_3_], 265, 249, 214, 206, 139 and 138. The ^1^H-NMR showed four aromatic protons. A downfield singlet at δ8.80 was attributed to N-H. The ester methyl protons resonated at δ3.81 as a 3H singlet. A three-proton singlet at δ1.92 was assigned to CH_3_-18. The H-17a appeared at δ3.09 as a doublet (*J *= 15 Hz) while another doublet (*J *= 15 Hz) at δ2.84 was assigned to the H-17b. The ^13^C-NMR gave a total of 21 carbon resonances. Two C=O peaks at δ168.65 and δ168.75 were observed.

Vincamine was isolated as white crystals, m.p. 230–233°C. The IR spectrum showed intense absorption bands at 3330 (O-H) along with other absorption bands at 1748 (ester C=O), 1615 (C=C), and 742 (1, 2-disubstituted benzene ring). The EI-MS showed a molecular ion at *m/z *354 with other ion fragments of 339 [M^+^-CH_3_], 325 [M^+^-C_2_H_5_], 307, 295 [M^+^-COOCH_3_], 284, 267, 252, 237, 224, 209, 180, 167, 149, 133 and 115. The ^1^H-NMR showed four aromatic protons. The ester methyl protons resonated at δ3.82 as a 3H singlet. A singlet at δ4.57 was assigned to 14-OH. The H-15a appeared at δ2.21 as a doublet (*J *= 15.1 Hz) while another doublet (*J *= 15.1 Hz) at δ2.11 was assigned to the H-15b. The one-proton singlet at δ3.92 was assigned to H-3 which was deshielded by two N atoms. The ^13^C-NMR gave a total of 21 carbon resonances. Ester C=O peak appeared at δ174.89.

Vincamine was observed in the retention time of 5.2 min in the HPLC chromatogram of the sample and was well separated. Quantization results showed 0.057% of vincamine in dried plant material (115 ± 5.64 μg/mL in the sample). The results obtained from HPLC method validation for vincamine assay according to linearity, selectivity, precision and accuracy showed that the proposed method was reliable. Excellent linearity was obtained between peak areas and concentrations 50, 100, and 200 μg/mL with *r*^2^ = 0.9995 for vincamine. Vincamine peaks in the sample and the standard chromatograms were spectrally similar and pure. LODs and LOQs were calculated as 0.85 and 2.58 μg/mL. The precision results for analysis of vincamine showed RSD% ≤ 2.1 for intra-day and SRD ≤ 4.9 for inter-day precision, which are reasonable. Accuracy, which was evaluated as recovery after spiking the sample with standards at two levels, was found to be 97.1%. A reported article on HPLC determination of alkaloids of *V. minor *collected from western Slovakia revealed that the major alkaloids of the leaves were vincamine (0.132% PM), 1, 2-dehydroaspidospermidine (0.060% PM), and vincaminoreine (0.028% PM) (20). The monomeric indole alkaloids vincaminorine and vincaminoreine display a strong cytotoxic effect on leukemia P388 cells (21). Vincamine was also the major alkaloid present in the plant cultivated in Iran. However, its yield can be enhanced by providing the specific requirements of this species. As the total content of alkaloids reaches 0.15-1.4% of plant dry matter, adequate selection of soil and agricultural practices, such as planting, fertilization, irrigation, harvest date *etc*, can have a drastic influence on alkaloid yield. It has been reported that the best dates for vegetative propagation of *V. minor *are early autumn and early spring and high yields can be obtained with a recommended density of 400,000-600,000 plants per hectare. The fertilization and soil management should be carried out at the planting stage. It has been shown that the yield of alkaloids in cultivated plants is several times higher, as compared to naturally grown plants (11). In general, the fertilization of medicinal plants causes an increase in the yield of bioactive compounds. Six macroelements (N, P, K, Mg, Ca, S) and seven microelements (Fe, Mn, Zn, B, Cu, Mo, Cl) are essential for the normal physiological processes of plants. An adequate mineral nutrition in the initial phase of plant growth and development is the most important (10). In case of *Vinca minor*, nitrogen is the main yield-determining factor, followed by other macroelements. Nitrogen with the dose of 100-150 Kg per hectare significantly increases vincamine and the total alkaloid content. Application of phosphorus oxide in the dose of 70 Kg per hectare is also profitable for higher yield of herb (11). A study on effects of environmental factors on *Catharanthus roseus *alkaloid yield revealed that nitrogen and Zinc play an important role in the synthesis of alkaloids; Zn is indispensable for the synthesis of tryptophan, which is the precursor of indole alkaloids, whereas N is a constituent of the alkaloids (10). Irrigation is also a determining factor in plant yield. Optimal average annual rainfall for *V. minor *should be about 600-800 mm. During plant growth and development, low soil and air moisture should be avoided. Finally, it has been reported that the best period for harvesting the herb is from August to September when the alkaloid content, especially vincamine, reaches its maximum amount (11).

In conclusion, all of the aforesaid factors should be taken into consideration in order to optimize the growth and alkaloid yield of the plant cultivated in Iran. Vincamine, the pharmacologically most valuable alkaloid of *V. minor *so far, shows pronounced cerebrovasodilatory activity and acts as a regulator of cerebral blood circulation. Vincamine and its derivatives are marketed by some pharmaceutical companies in Europe and Japan as a cerebral vasodilator (22). Considering the importance of this plant and its major alkaloid, vincamine, it can be introduced to the pharmaceutical industry as a natural source for pharmaceutical preparations.
